# Polarity reversal of canine intestinal organoids reduces proliferation and increases cell death

**DOI:** 10.1111/cpr.13544

**Published:** 2023-09-11

**Authors:** Georg Csukovich, Maximilian Wagner, Ingrid Walter, Stefanie Burger, Waltraud Tschulenk, Ralf Steinborn, Barbara Pratscher, Iwan Anton Burgener

**Affiliations:** ^1^ Department for Companion Animals and Horses, Small Animal Internal Medicine Vetmeduni Vienna Austria; ^2^ VetBioBank, VetCore Vetmeduni Vienna Austria; ^3^ Institute of Morphology, Working Group Histology Vetmeduni Vienna Austria; ^4^ Genomics Core Facility, VetCore Vetmeduni Vienna Austria

## Abstract

Apical‐out intestinal organoids are a relatively simple method of gaining access to the apical cell surface and have faced increasing scientific interest over the last few years. Apical‐out organoids can thus be used for disease modelling to compare differing effects on the basolateral versus the apical cell surface. However, these ‘inside‐out’ organoids die relatively quickly and cannot be propagated as long as their basal‐out counterparts. Here, we show that apical‐out organoids have drastically reduced proliferative potential, as evidenced by immunohistochemical staining and the incorporation of the thymidine analogue EdU. At the same time, cell death levels are increased. Nevertheless, these phenomena cannot be explained by an induction of differentiation, as the gene expression of key marker genes for various cell types does not change over time.

## Graphical abstract

Basal out organoid polarity can easily be reversed by extracellular matrix removal to generate fully apical out intestinal organoids. However, this polarity reversal is accompanied by decreased proliferation coupled with increased cell death, ultimately leading to the death of apical out organoids, questioning their applicability and usefulness for disease modelling. Despite these differences, we demonstrate that transcript expression of key intestinal marker genes is unaffected by polarity reversal.

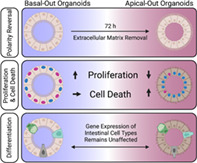

## 1 INTRODUCTION

Gastrointestinal diseases represent a major health burden all over the world. One of the most important gastrointestinal diseases in humans is inflammatory bowel disease (IBD) with an estimated 6.8 million people living with IBD.^1^ Interestingly, IBD does not only occur in humans but also affects animals like dogs and cats, but it is not a naturally occurring disease in common laboratory rodents.^2^ Therefore, gastrointestinal diseases do not only cause immensely high costs for health care systems but also to pet owners and require in‐depth analysis as commonly used treatments are unsatisfactory and the underlying cause for IBD is multifactorial and still not entirely known.^3^

In the search of developing new in vitro model systems that resemble physiological properties found in vivo, organoids have been around for almost 15 years since the first establishment of intestinal organoids from *Lgr5* ^+^ stem cells.^4^ In the meantime, organoids comprise a variety of different organs, including several gastrointestinal ones like stomach,^5^ liver^6^ and pancreas,^7^ besides the classical intestinal organoids. The complex three‐dimensional architecture of organoids, which consist of several different cell types (e.g., stem cells, enterocytes, goblet cells and enteroendocrine cells), can be established from either adult stem cells or induced pluripotent stem cells (iPSCs) via targeted differentiation. In this regard, canine intestinal organoids are not only necessary for veterinary research, but could also potentially replace live dogs used for research (especially pharmacological testing) in the future.

However, intestinal organoids bear the major drawback of the apical cell surface of epithelial cells being inaccessible and hidden on the inner surface of the organoids. Han et al. provide a very good review on the possibilities one has to access the apical surface.^8^ Apart from the possibility to physically disrupt organoids to reach the apical side temporarily, generating organoid‐derived monolayers^9^ or using microinjection techniques^10^ have become alternative approaches. Another feasible method is the generation of so‐called ‘apical‐out’ organoids. In regard to this method, we have previously reviewed different disease modelling approaches using intestinal organoids, especially in a One Health context.^11^

Apical‐out organoids were first published by Co et al., who have demonstrated that human intestinal organoids that usually grow embedded in a three‐dimensional extracellular matrix (ECM) can reverse their polarity when they are cultured in a floating manner in medium without ECM.^12^ This method has now been adopted by many other labs and expanded to different animal species, for example, pigs,^13^ chickens,^14^ cows^15^ and dogs,^16^ and also organoids derived from human iPSCs and embryonic stem cells.^17^, ^18^ Remarkably, regardless of the species from which the organoids were derived, the majority of these studies utilised their apical‐out organoids within the initial 7 days following the induction of polarity reversal.^13^, ^16^, ^17^, ^19^, ^20^, ^21^ Experiences from our lab show, that canine intestinal apical‐out organoids produce a lot of cell debris within the culture well, which is most probably due to dying cells being extruded from the epithelial layer of cells directly into the medium. This phenomenon increases until apical‐out organoids slowly start to die off completely after approximately 1 week of culture. Taken together, these observations lead to the hypothesis that the polarity reversal of intestinal organoids leads to a change in proliferative behaviour and a higher rate of apoptotic cells in apical‐out organoids compared to basal‐out organoids.

According to previous reports, whether the induction of polarity reversal is associated with organoid differentiation is to some extent controversial. Two studies show that apical‐out organoids do not present immensely different gene expression profiles compared to their basal‐out counterparts as long as they are cultured in their standard growth medium.^12^, ^18^ However, according to another study using porcine organoids, the expression of the stem cell marker termed leucine‐rich repeat‐containing G‐protein coupled receptor 5 (*LGR5*) is diminished after 3 days of apical‐out culture and differentiation markers chromogranin A (*CHGA*; enteroendocrine cells), mucin‐2 (*MUC2*; goblet cells) and intestinal alkaline phosphatase (*ALPI*; enterocytes) show strikingly elevated expression.

In this study, we successfully established apical‐out intestinal organoids from canine small and large intestines and quantified the rate at which organoids reverse their polarity after ECM removal. Additionally, we show that apical‐out organoids have drastically decreased number of actively proliferating cells after 36 h. We then analysed organoid viability and cell death in basal‐out and apical‐out organoids over a period of 72 h and discovered that apical‐out organoids have lower cell viability‐coupled with higher cell death rates, which might explain why apical‐out organoids die after a few days in culture. However, reverse transcription‐quantitative polymerase chain reaction (RT‐qPCR) data show that this increase in cell death does not coincide with increased differentiation in canine intestinal organoids.

Overall, we would like to emphasise that newly established model systems require careful characterisation before their utilisation as certain attributes can fundamentally influence outcomes and potentially skew findings.

## 2 RESULTS

### 2.1 Polarity reversal

**FIGURE 1:**
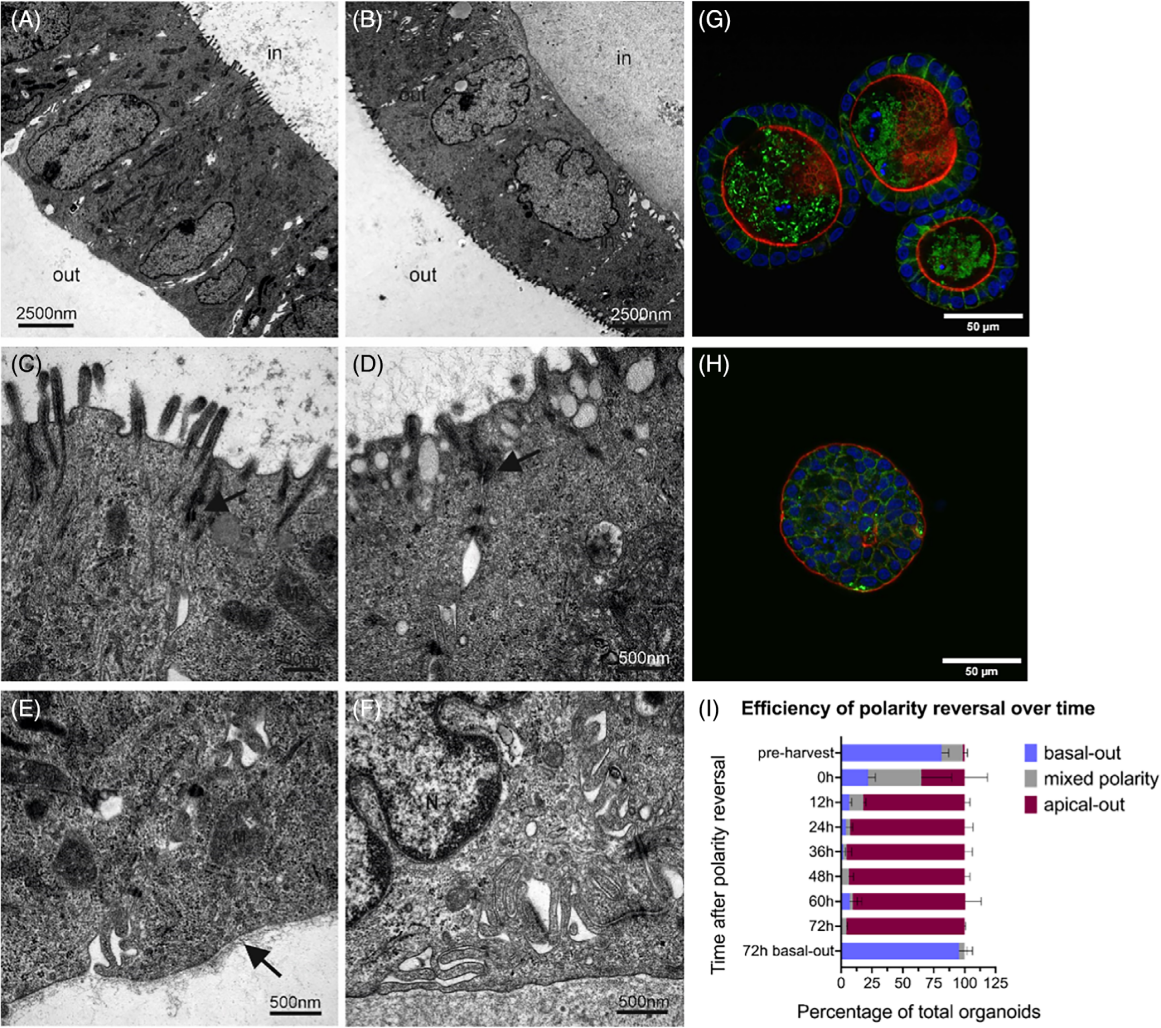
Polarity reversal of canine intestinal organoids. (A–F) Electron microscopic images representing basal‐out (A, C, E) and apical‐out (B, D, F) organoids. Arrows indicate desmosomes in (C) and (D) and a basal lamina in (E). M = mitochondria; N = nucleus. Immunofluorescent stainings of basal‐out (G) and apical‐out (H) organoids with Claudin‐7 (green) and Phalloidin (red) representing the flipped polarity. Nuclei were stained with DAPI (blue). (I) Quantitative analysis of organoid repolarisation over time. Data are presented as mean ± standard deviation.

We quantified the efficiency of polarity reversal over time, in intervals of 12 h, after inducing polarity reversal. This quantification was based on DAPI/Phalloidin stainings of organoids as can be seen in Figure 1G,H. Successful polarity reversal was also evidenced by transmission electron microscopy (TEM) of basal‐out and apical‐out organoids (Figure 1A–F). While basal‐out organoids present their apical microvilli into the organoid lumen, apical‐out organoids are oriented in the other direction. Organoids feature apical desmosomes and a basal lamina, as seen in TEM images. Unexpectedly, nearly 40% of organoids exhibited apical‐out polarity at the onset of the polarity reversal (Figure 1I), with the majority of the remaining organoids displaying mixed polarity. This was despite the fact that the vast majority of organoids show a basal‐out state before ECM removal. However, we could verify that 72 h after inducing polarity reversal, almost 100% of organoids are apical‐out with a small number of mixed polarity organoids and absolutely no basal‐out oriented organoids. On the other hand, floating basal‐out controls remain in a basal‐out state 72 h after harvest.

**FIGURE 2:**
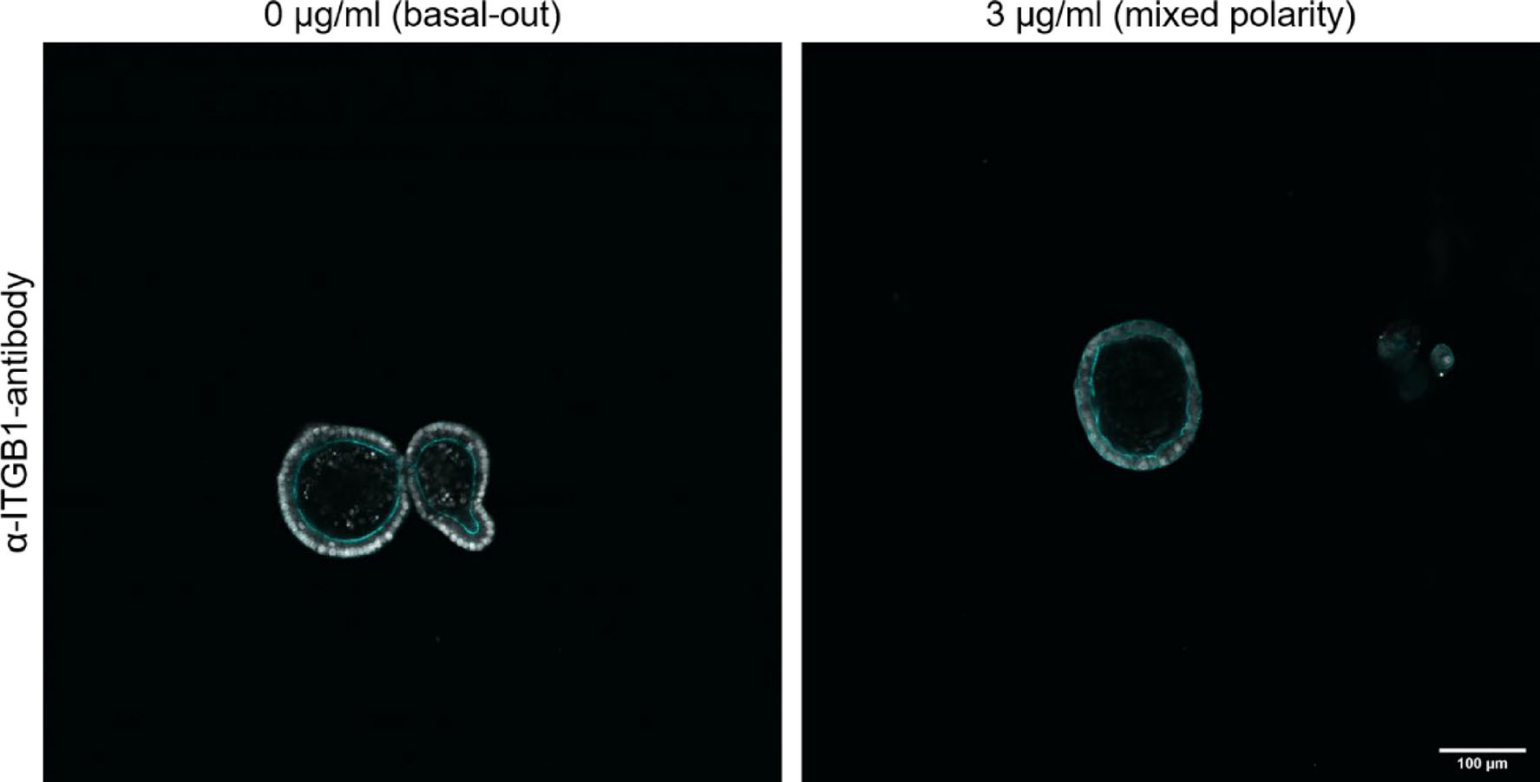
Organoids incubated with α‐ITGB1‐antibody for 3 days partially reverse their polarity. In contrast to control organoids (left), which remain in a basal‐out state, ITGB1‐antibody treated organoids present a mixed polarity state in which Actin staining (cyan) indicative of microvilli can be seen on both surfaces of the organoids. Nuclei were stained with Hoechst (grey).

Treatment with α‐ITGB antibody at a concentration of 3 μg/mL for 3 days led to mixed polarity organoids, with no full polarity reversal (Figure 2). Further increasing the concentration of α‐ITGB merely led to an increase in cell death. Thus, attempting to reverse the polarity of canine intestinal organoids with α‐ITGB is not advisable and does not yield efficiently re‐polarised organoids for downstream experiments.

### 2.2 EdU staining

**FIGURE 3:**
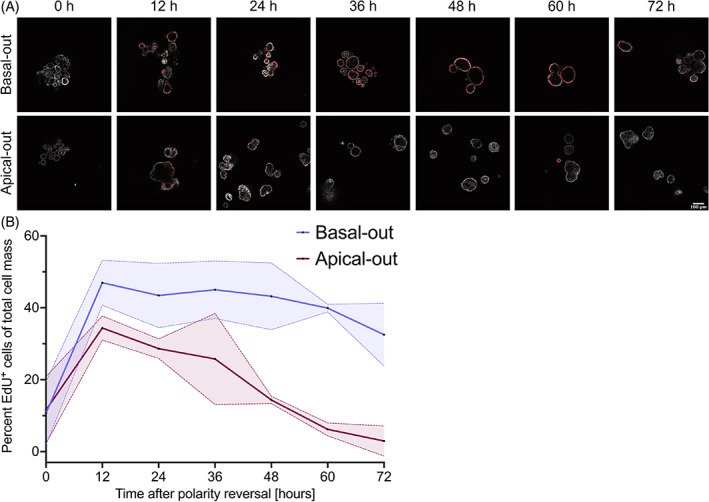
(A) Staining of EdU incorporation (red) in small intestinal basal‐out and apical‐out canine intestinal organoids over 72 h, highlighting the reduced proliferation of apical‐out organoids compared to their basal‐out counterparts. Nuclei were stained with Hoechst (grey). (B) Quantification of EdU^+^ cells of both small and large intestinal basal‐out and apical‐out organoids. There is a significant interaction effect of time × polarity (*p* value 0.0028).

To get an overview of the proliferation status of organoids after polarity reversal, we incubated organoids with 10 μM EdU for 1.5 h every 12 h starting at the time point when polarity reversal was induced. While proliferation, as measured by EdU^+^ cells, was high right until the end at 72 h in basal‐out organoids, apical‐out organoids showed a clear reduction of proliferating cells. The decrease in proliferation was most pronounced comparing the 24‐h time point to the 36‐h time point after polarity reversal, where we noticed a clear drop of EdU^+^ cells, exemplified by data from small intestinal organoids (Figure 3A). A very similar pattern can also be seen in organoids from the large intestine (Figure S1). Image analysis using Arivis4D allowed us to quantify the amount of EdU^+^ cells over time, highlighting the clear difference between basal‐out and apical‐out organoids with a significant interaction effect of time × polarity (*p* value 0.0028).

Although treatment with α‐ITGB antibody was unsuccessful in inducing complete polarity reversal, integrin signalling will be reduced in apical‐out organoids due to the absence of ECM, with the lack of signalling ultimately impacting the MAPK pathway.^22^, ^23^ Therefore, we attempted to re‐activate MAPK‐signalling using the small‐molecule activator Senkyunolide I (SENI), an agonist of Erk1/2. However, boosting MAPK signalling did not lead to higher numbers of EdU^+^ cells in apical‐out organoids after 3 days of SENI treatment compared to untreated controls (Figure S2).

### 2.3 Viability and cell death

**FIGURE 4:**
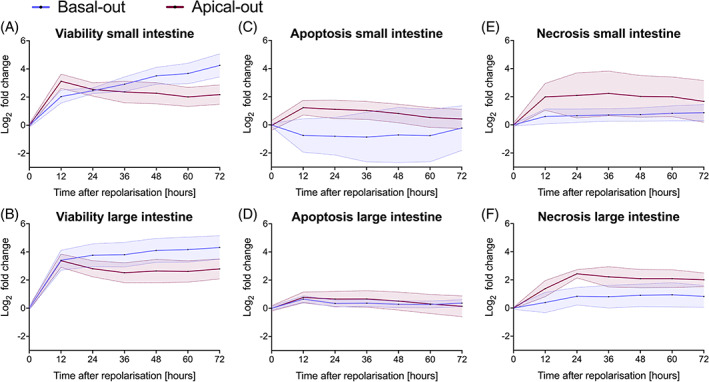
Viability, apoptosis and necrosis measurements of small and large intestinal organoids. The effect of polarity was significant over 72 h in all samples according to a two‐way ANOVA. Data are presented as mean ± standard deviation of three different small and large intestinal organoids, which were measured in eight individual reactions each. ANOVA, analysis of variance.

After noticing that organoids seemingly stop proliferating after reversing their polarity, we used viability and apoptosis/necrosis assays for further analysis of the difference between basal‐out and apical‐out organoids. The viability assay, serving as an indicator of cell mass, reveals that basal‐out organoids continue to increase their cell mass, that is, proliferate, until the final time point at 72 h following polarity reversal. However, apical‐out organoids only show a small peak at 12 h post‐polarity reversal with subsequently decreasing viability values. This is consistent with apoptosis and necrosis measurements, in which apical‐out organoids generally present slightly higher values than basal‐out organoids. Overall, all samples show highly significant values when analysing the difference between basal‐out to apical‐out organoids over a time course of 72 h, except for colonic apoptosis, where polarity did not have a significant effect (Figure 4 and Table S1).

**FIGURE 5:**
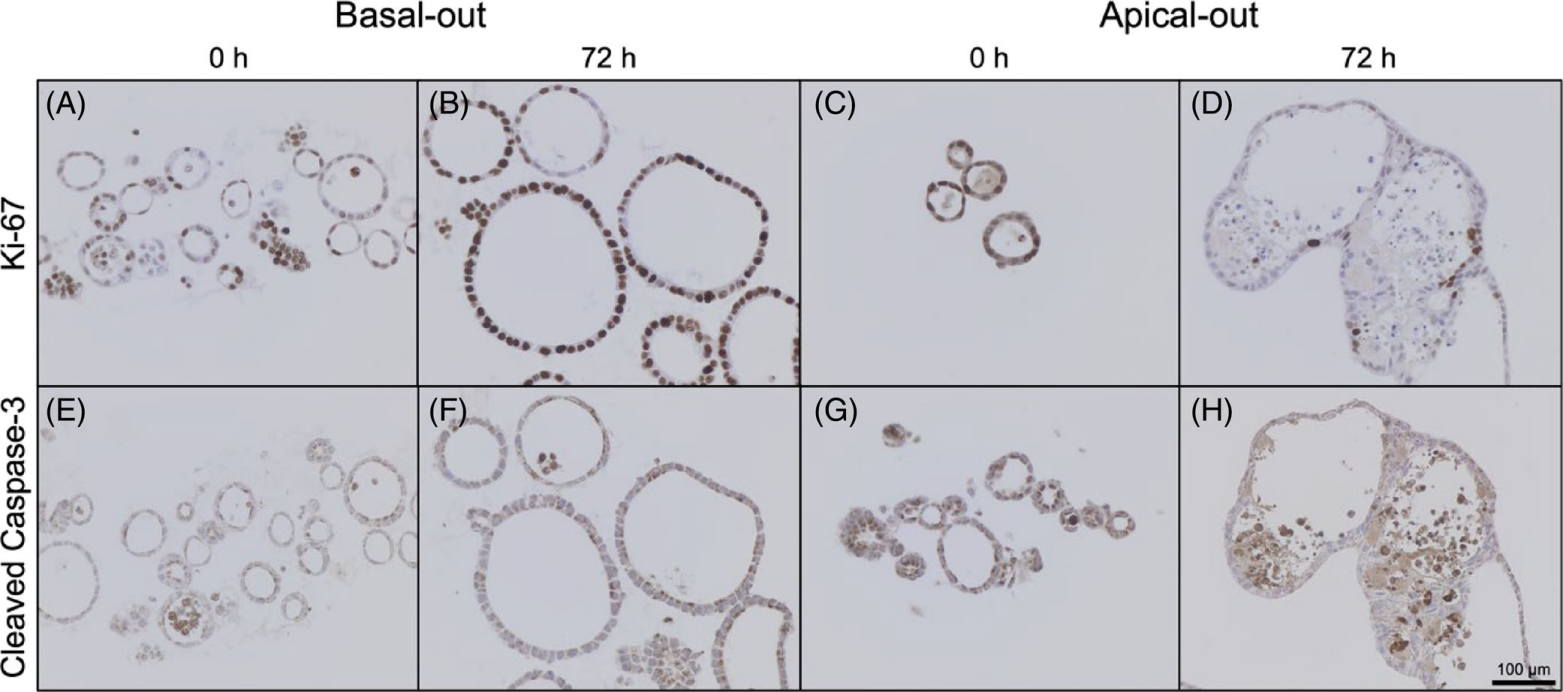
Immunohistochemical stainings of basal‐out and apical‐out small intestinal organoids for proliferation marker Ki‐67 and apoptosis marker cleaved caspase‐3. Scale bar = 100 μm.

These data were further verified with immunohistochemical staining of Ki‐67 (for proliferation) and cleaved caspase‐3 (for apoptosis) in both small intestinal (Figure 5) and large intestinal organoids (Figure S3). Immunohistochemical stainings fit very well to the aforementioned data of higher proliferation in basal‐out organoids and slightly increased cell death in apical‐out organoids. While all organoids show similar properties at time point 0, basal‐out organoids are steadily increasing in size, cell number and the amount of Ki‐67^+^ cells. Apical‐out organoids generally appear more unstructured with the single epithelial layer being less clear at 72 h after polarity reversal (see also Figure 1H). Especially areas in the centre of apical‐out organoids show more cleaved caspase‐3^+^ cells.

### 2.4 Transcript expression

**FIGURE 6:**
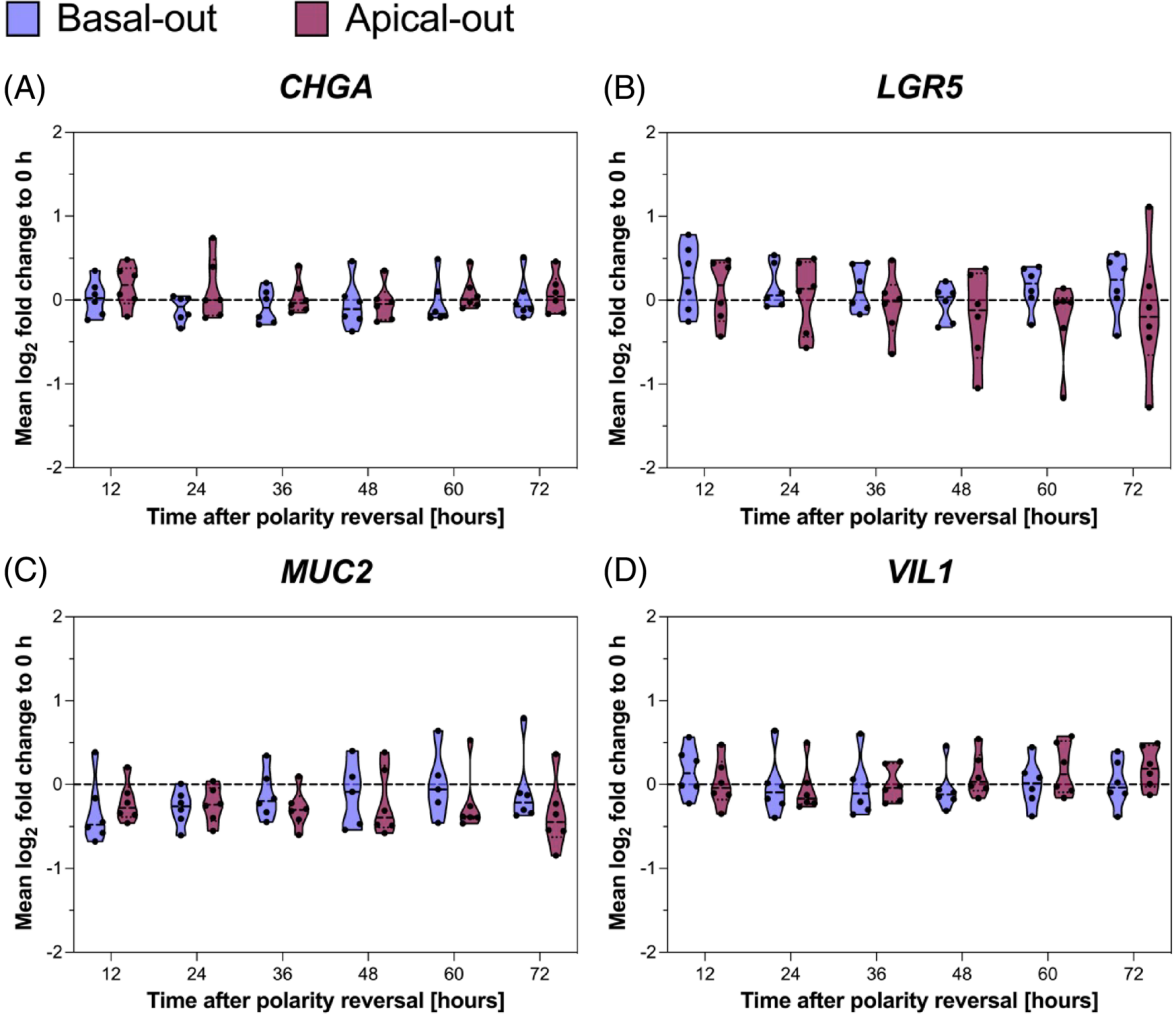
RT‐qPCR analysis of basal‐out and apical‐out organoids after inducing polarity reversal. Only minor changes in transcript abundance can be observed indicating stability of culture condition. Normaliser: geometric mean of *DAP3* and *ESD*, calibrator: time point of 0 h (not depicted). *n* = 6 (three small and three large intestinal organoids). RT‑qPCR, reverse transcription‐quantitative polymerase chain reaction.

One possible explanation for reduced proliferation concomitant with increased apoptosis could be increased cell differentiation leading to stem cell exhaustion. To define whether cell differentiation after polarity reversal is responsible for reduced proliferation in apical‐out organoids, we collected organoids every 12 h after removing the ECM to perform RT‐qPCR analysis. Compared to directly after ECM removal (i.e., 0 h time point), there are no significant changes in transcript expression of the key stem cell marker gene *LGR5* or markers for cell type differentiation (*CHGA, MUC2* and *VIL1*) over time. However, there is a trend towards *LGR5* and *MUC2* transcript reduction (Figure 6).

## 3 DISCUSSION

The efficiency of polarity reversal was very comparable to previously published data with almost no basal‐out organoids left after 36–48 h after inducing repolarisation,^12^ and seemed to be much more efficient than porcine intestinal organoids, where only about 40% of organoids are in an apical‐out state after 48 h.^13^ However, astonishingly only after incubation with organoid harvesting solution for 1.5 h, about two thirds of all organoids showed apical‐out or mixed polarity based on phalloidin staining. Considering that before the utilisation of organoid harvesting solution, over 80% of organoids exhibited basal‐out polarity, it really is remarkable that organoids are capable of rapidly reversing their polarity, even in cold buffered harvesting solution where cellular functions, such as cytoskeletal remodelling, would be expected to operate less efficiently than under normothermic conditions. Even though many organoids do not show basal‐out polarity directly after ECM harvest, almost 100% of organoids are in a basal‐out state when cultured in medium supplemented with 7.5% Geltrex. Thus, two different scenarios are possible: initially seeded apical‐out and mixed polarity organoids could potentially die in basal‐out medium and only basal‐out organoids remain at 72 h post‐harvest or apical‐out organoids return to a basal‐out state. These findings suggest that selecting the appropriate time point for subsequent experiments may be critical, as the polarity of organoids could impact the results and the utilisation of mixed polarity organoids may not yield meaningful data.

Even though canine intestinal organoids appear to be closer to human than porcine organoids in terms of repolarisation efficiency, we could not reverse organoid polarity using the same α‐ITGB1 antibody as Co et al.^12^ Even though organoid polarity changed to a more mixed‐polarity state, no organoids presented complete apical‐out characteristics after being treated with α‐ITGB1 for 3 days. Consequently, in the case of canine intestinal organoids, β1‐integrins do not appear to be the sole determinant of proper organoid polarity, and their inhibition does not induce complete polarity reversal, as opposed to human organoids. The exact mechanism for organoid polarity inversion in canine intestinal organoids remains unexplained and will have to be explored more in‐depth in follow‐up studies. Nevertheless, integrin signalling can potentially influence many other intracellular signalling pathways, for instance, the MAPK signalling pathway, thus directly affecting proliferation, differentiation and cell death. However, the attempt to activate MAPK signalling via activation of Erk1/2 using small‐molecule activator SENI was not successful, with very few EdU^+^ cells in apical‐out organoids, as is the same in untreated organoids. Therefore, we propose that other factors, different from MAPK signalling, determine whether organoids stop proliferating upon polarity reversal.

Viability and cell death measurements make clear, why apical‐out organoids die after about 1 week. While basal‐out organoids show constantly increasing viability values, apical‐out organoid viability rates drop after an initial peak at 12 h after polarity reversal. Together with apoptosis and necrosis values, which are both generally higher in apical‐out organoids than in basal‐out organoids, this leads to a decreasing number of cells. This indicates that more apoptotic/necrotic cells coupled with a smaller number of proliferating cells, ultimately lead to dying organoids. These results are further validated by Ki‐67 (i.e., proliferation) and cleaved caspase‐3 (i.e., apoptosis) immunohistochemical stainings. These stainings highlight the significantly higher proliferative potential of basal‐out organoids over apical‐out organoids. Concurrently, there are fewer cleaved caspase‐3^+^ cells in basal‐out organoids, leading to much higher viability in basal‐out compared to apical‐out organoids.

In general, intestinal organoids are very stable culture systems over many passages.^24^, ^25^ Despite this, there exist conflicting data on cell differentiation in apical‐out organoids. In two studies, stem cell markers (*LGR5* and *ASCL2*) remain constant or even increase in expression. Similarly, *CHGA* and *MUC2* show no change. Interestingly, Co et al. show increased expression of enterocyte marker gene sucrose isomaltase (*SI*), while Kakni et al. demonstrate slightly decreasing levels of enterocyte marker gene villin‐1 (*VIL1*).^12^, ^18^ Using our canine organoids, we demonstrate that overall, key marker genes are relatively stably expressed in our basal‐out and apical‐out organoids. While *LGR5* and *MUC2* expression decreases slightly, but non‐significantly, *VIL1* expression is slightly increased 72 h after polarity reversal. These data are partly in conflict with previous studies, too. We see a slight decrease in *LGR5* and *MUC2* levels but a slight increase in *VIL1* expression in canine organoids. However, since we analysed all samples every 12 h after polarity reversal, we can successfully rule out time‐dependent oscillations that might lead to random results and prove the high stability of our system in terms of gene expression. The reasons why other studies might deliver different results are manifold and range from differently composed culture media to different gene regulation in other species and the choice of appropriate reference genes for RT‐qPCR data normalisation.

Since polarity reversal shows drastic effects in terms of proliferation, this can have an immense effect on potential disease modelling applications. However, there is no obvious difference between small and large intestinal organoids as organoids from both sections of the intestine change in the same way upon polarity reversal. Given the fact that proliferation may affect various signalling pathways, basal‐out and apical‐out organoids might be different in many other ways. Therefore, reversing the polarity can not only change the morphology but also signalling within the cell and hence how organoids react to anything you intend to test, whether it is simply a change in medium components or something more complex like toxins or even pathogens like viruses or bacteria. Thus, any system using apical‐out organoids should be carefully assessed before use, especially when comparisons to basal‐out organoids are made. Our data show that it is critical to choose the correct time point for any experiment to minimise differences between basal‐out and apical‐out organoids.

## 4 CONCLUSION

Given the important differences between basal‐out and apical‐out organoids within this paper, it must be critically evaluated, whether apical‐out organoids can and should be used as a relevant in vitro model. Apical‐out organoids undeniably present crucial advantages over basal‐out organoids in some respects as the apical cell surface is directly accessible and therefore the epithelium can be easier challenged with all sorts of different toxins/pathogens that usually affect the intestine from its luminal surface. However, apical‐out organoids show much lower proliferation and viability and are not as long‐lived compared to basal‐out organoids despite presenting necessary stability in terms of gene expression and differentiation. Therefore, other options (e.g., organ‐on‐chip technology) might seem more tedious and costly, but could potentially be more relevant if the basolateral and apical cell surfaces are accessible at the same time.

This leads to the conclusion that the characteristics of apical‐out organoids should be critically evaluated before using them for disease modelling or similar approaches, especially if they are subject to comparisons to basal‐out organoids. Other options to gain access to the apical cell surface of intestinal organoids should be considered to support findings from studies using apical‐out organoids. For instance, organoid fragmentation, microinjection and the generation of organoid‐derived monolayers are valid alternative methods that can complement apical‐out studies.^8^

## 5 MATERIALS AND METHODS

### 5.1 Organoid culture

Canine intestinal crypts were isolated from jejunum and colon of three different dogs according to Kramer et al.^26^ Based on the guidelines of the institutional ethics committee, the use of tissue material collected during therapeutic excision or post‐mortem is included in the university's ‘owner's consent for treatment’, which was signed by all patient owners. Organoid growth medium consisted of 37% basal medium (Advanced DMEM/F12 supplemented with 2 mM GlutaMAX and 10 mM HEPES), 1 × B27 (Invitrogen, Thermo Fisher Scientific), 1 mM N‐acetylcysteine, 10 nM Gastrin (Sigma‐Aldrich), 100 ng/mL Noggin, 500 nM A8301, 50 ng/mL HGF, 100 ng/mL IGF1, 50 ng/mL FGF2 (PeproTech), 10% (v/v) Rspondin1 and 50% (v/v) Wnt3a conditioned media. For the first 2 days of culture, 50 ng/mL mEGF (Thermo Fisher Scientific) and 10 μM Rock‐inhibitor Y‐27632 (Selleck Chemicals) were added. The growth medium was changed every 2–3 days. Weekly passaging at 1:4 to 1:8 split ratios was achieved by mechanical disruption using flame‐polished Pasteur pipettes. For experiments where it is indicated, small‐molecule inhibitor Senkyunolide I (SENI) was used in a concentration of 5 μM as described previously.^27^, ^28^

### 5.2 Polarity reversal

Apical‐out and floating basal‐out organoids were generated as described previously.^12^ Organoids were harvested using Cultrex® Organoid Harvesting Solution (Bio‐Techne) for 1.5 h at 4°C, under constant shaking. Thereafter, organoids were washed with basal medium, resuspended in growth medium and seeded in multiwell plates treated with Anti‐Adherence Rinsing Solution (Stemcell Technologies) to prevent organoid attachment to the surface. To generate floating basal‐out organoids, 7.5% Geltrex (Thermo Fisher Scientific) was added to the culture medium. Organoids were incubated for up to 72 h in a humidified incubator with 5% CO_2_ before further use.

### 5.3 Transmission electron microscopy

To further analyse polarity reversal of organoids, basal‐out and apical‐out organoids at day three after induction of polarity reversal were used. All samples were fixed in 3% buffered glutaraldehyde (pH 7.4, Merck). Organoids were then pre‐embedded in 1.5% agarose. After being washed in 0.1 M Soerensen buffer (pH 7.4), the samples were postfixed for 2 h at room temperature in 1% osmium tetroxide (Electron Microscopy Sciences). This was followed by dehydration in an ethanol series along with an increasing series of propylene oxide (Sigma‐Aldrich) before embedding and polymerisation in epoxy resin (Serva) for 48 h at 60°C. Ultrathin sections (70 nm) were cut for transmission electron microscopic evaluation and contrasted in methanolic uranyl acetate (Fluka Chemie AG) and alkaline lead citrate (Merck). For imaging, a transmission electron microscope (EM 900, Zeiss) equipped with a slow‐scan CCD camera (2k Wide‐angle Dual Speed, TRS) and ImageSP Professional software (SYSPROG, TRS) were used.

### 5.4 Immunofluorescent staining

Organoids were fixed with 2% (v/v) paraformaldehyde (PFA) and stained according to a previously published protocol including a clearing step after organoid staining.^29^ Organoids were stained with 1:100 Claudin 7 Polyclonal Antibody (Invitrogen, Thermo Fisher Scientific) for tight junctions with secondary antibody AF‐488 goat anti‐rabbit (Invitrogen, Thermo Fisher Scientific) diluted 1:500, 1:200 phalloidin (Alexa Fluor 647, Invitrogen, Thermo Fisher Scientific) to visualise actin filaments, and with 4 μg/mL 4′,6‐diamino‐2‐phenylindole (DAPI; Sigma‐Aldrich) for nuclear staining. Confocal images were acquired using a Zeiss LSM 880 confocal microscope (Zeiss).

### 5.5 EdU staining

To assess cell proliferation, the Click‐iT® EdU Imaging Kit (Invitrogen, Thermo Fisher Scientific) was used. Basal‐out and apical‐out organoids of the small and large intestine were incubated with 5‐ethynyl‐2′deoxyuridine (EdU) at a final concentration of 10 μM for 1.5 h at 37°C and were then fixed with 2% (v/v) PFA for 15 min at room temperature. Staining was carried out according to the manufacturer's instructions. DNA was counterstained using Hoechst33342 (Abcam). Confocal images were taken using an LSM 880 (Zeiss). The acquired images were further analysed using the Vision4D Software by Arivis/Zeiss. Using this software, organoids chosen for analysis were encircled individually and segmented into their nuclei positive for Hoechst and EdU, respectively. Only nuclei larger than 15 μm^2^ were considered for the analysis. A total number of 390 organoids were analysed. Dead cells within the organoid lumen were excluded. After calculating the area of the positive cell nuclei, we summed up all the numbers for each time point at both polarity states before calculating the percentage of EdU^+^ cells of the total cell mass (EdU^+^ and Hoechst^+^ nuclei). This number then serves as an estimate of EdU^+^ proliferating cells within our sample.

### 5.6 Viability and cell death assays

Viability and apoptosis of basal‐out and apical‐out organoids were assessed using the RealTime‐Glo MT Cell Viability Assay (Promega) and RealTime‐Glo Annexin V Apoptosis Assay (Promega; referred to as ‘apoptosis and necrosis assay’). Equal numbers of organoids were seeded into each well of a white 96‐well plate with clear bottom to induce polarity reversal as described above. Detection reagents were prepared according to the manufacturer's instructions and added to the respective wells. Luminescence was measured at 0, 12, 24, 36, 48, 60 and 72 h after adding the substrates at time point 0 h using a GloMax Explorer plate reader (Promega). Experiments were carried out in eight technical replicates of three biological replicates per intestinal section (small and large intestines).

### 5.7 Immunohistochemistry

Organoids were fixed in 2% (v/v) PFA for 15 min and embedded in paraffin. Sections from paraffin tissue blocks were cut for standard immunohistochemical staining. All slides were deparaffinised with xylene and rehydrated through a graded series of alcohols followed by endogenous peroxidase blocking with 0.6% hydrogen peroxide in methanol. Heat retrieval in citrate buffer (pH 6.0) was done with a steamer. Protein blocking was performed with 1.5% goat serum (Sigma‐Aldrich) in PBS. Immunolabelling was performed by incubation with a monoclonal mouse anti‐Ki‐67 antibody (MIB1, DAKO, #M724029) at a dilution of 1:500 in PBS or a monoclonal rabbit anti‐cleaved caspase‐3 antibody (#9664, Cell Signaling Technology) at a dilution of 1:250 in PBS overnight at 4°C followed by 60 min at room temperature. The next day, sections were incubated with the respective horseradish peroxidase (HRP) labelled secondary antibody (mouse or rabbit Immunologic Bright Vision HRP) and the signal was detected by 3′3′diaminobenzidine (Bright DAB, Immunologic, Arnhem) reaction. ‘No primary antibody’ controls were used to show that there was no host‐specific binding of the secondary antibodies.

### 5.8 Reverse transcription‐quantitative polymerase chain reaction

At time points 0, 12, 24, 36, 48, 60 and 72 h after induction of polarity reversal, basal‐out and apical‐out organoids were harvested. Organoid RNA was isolated using the ReliaPrep RNA Tissue Miniprep System according to the manufacturer's instructions (Promega). About 500 ng RNA was subjected to RT with oligo‐dT and random hexamer primers according to the manufacturer's recommendations (GoScript Reverse Transcription System, Promega). Dye‐based qPCR was carried out using GoTaq® qPCR Master Mix (Promega) and the primer sequences were provided in Table S2. Amplification conditions were as follows: 15 min of initial denaturation at 95°C, 40 cycles of 15 s of denaturation at 95°C, 60 s of annealing/extension at 60°C and a read step, followed by 10 s of dissociation at 95°C and a melting curve from 65°C to 95°C in 5 s per 0.5°C increments. Quantitative data analysis involved adjustment of experimental amplification efficiency (*E*).^30^ The efficiency of each individual sample was calculated in silico from non‐baseline‐corrected fluorescence values using the Real‐time PCR Miner software^31^ (http://miner.ewindup.cn/miner/). Experimentally measured *Cq* values were adjusted by the term *Cq* × (log_10_(*E* + 1)/log_10_(2)).^32^ Outlying triplicates of samples causing a standard deviation of more than 0.5 were excluded from analysis. Abundance of a target transcript was normalised to the geometric mean of the reference‐gene pair *DAP3* and *ESD* (manuscript in preparation) and subsequently calibrated to the 0 h time point and presented as mean log_2_ fold‐change.

### 5.9 Statistical analysis

Data from EdU staining and log‐transformed data from viability, apoptosis and necrosis measurements as well as RNA expression data were subjected to statistical analysis by means of a two‐way analysis of variance to take time and polarity into account. Statistical evaluation was performed using GraphPad Prism 9 (GraphPad Software).

## AUTHOR CONTRIBUTIONS

*Conceptualization*: Georg Csukovich, Maximilian Wagner and Barbara Pratscher. *Methodology*: Georg Csukovich, Ingrid Walter and Ralf Steinborn. *Formal analysis*: Georg Csukovich and Maximilian Wagner. *Investigation*: Georg Csukovich, Maximilian Wagner, Ingrid Walter, Stefanie Burger and Waltraud Tschulenk. *Data curation*: Georg Csukovich, Maximilian Wagner, Ingrid Walter. *Writing—original draft*: Georg Csukovich and Maximilian Wagner. *Writing—review and editing*: Georg Csukovich, Maximilian Wagner, Ingrid Walter, Stefanie Burger, Waltraud Tschulenk, Ralf Steinborn and Stefanie Burger. *Visualisation*: Georg Csukovich, Maximilian Wagner, Ingrid Walter, Stefanie Burger and Waltraud Tschulenk. *Supervision*: Georg Csukovich, Barbara Pratscher and Iwan Anton Burgener. *Funding acquisition*: Georg Csukovich and Iwan Anton Burgener.

## FUNDING INFORMATION

GC is a recipient of a DOC fellowship (grant number 26349) of the Austrian Academy of Sciences (ÖAW) at the Division for Small Animal Internal Medicine at Vetmeduni.

## CONFLICT OF INTEREST STATEMENT

The authors declare no conflicts of interest.

## Supporting information


**DATA S1.** Supporting Information.

## Data Availability

All relevant data can be found within the article and its Supporting Information. Additional raw data supporting the conclusions of this article will be made available by the authors upon inquiry.
