# Supplementary material for: Polarity reversal of canine intestinal organoids reduces proliferation and increases cell death
Source: Cell Prolif. 2023 Sep 11;57(2):e13544. doi: 10.1111/cpr.13544 (PMC10849783; doi:10.1111/cpr.13544)
Supplement: Supplementary file 1 — DATA S1. Supporting Information. [file CPR-57-e13544-s001.docx]

# SUPPLEMENT


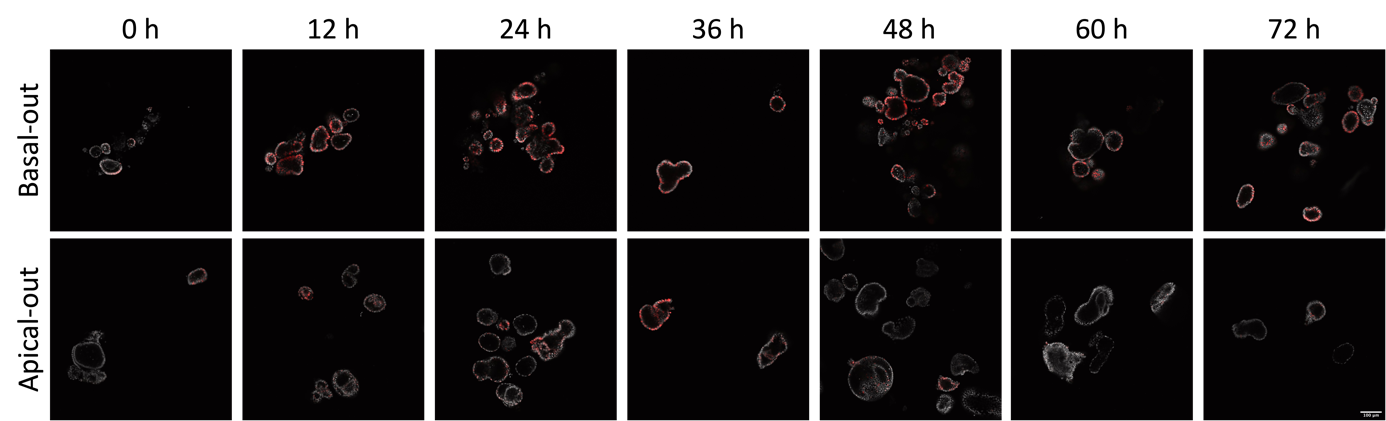


Supplementary figure 1: Staining of EdU incorporation (red) in large intestinal basal-out and apical-out canine intestinal organoids over 72 hours, highlighting the reduced proliferation of apical-out organoids compared to their basal-out counterparts. Nuclei were stained with Hoechst (grey).


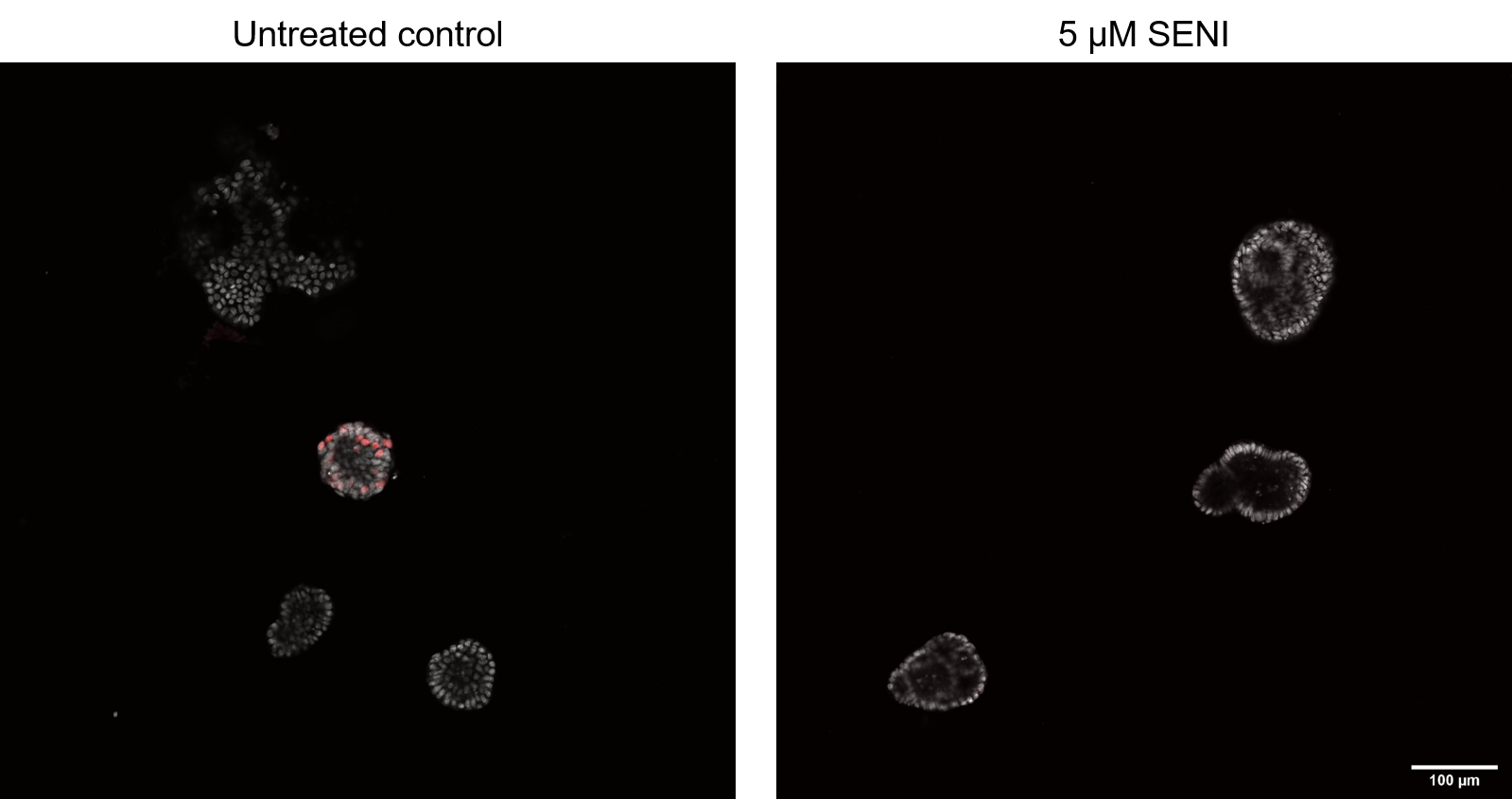


Supplementary figure 2: Apical-out organoids treated with 5 µM Senkyunolide I for 72 hours do not show a significantly elevated number of EdU^+^ cells.


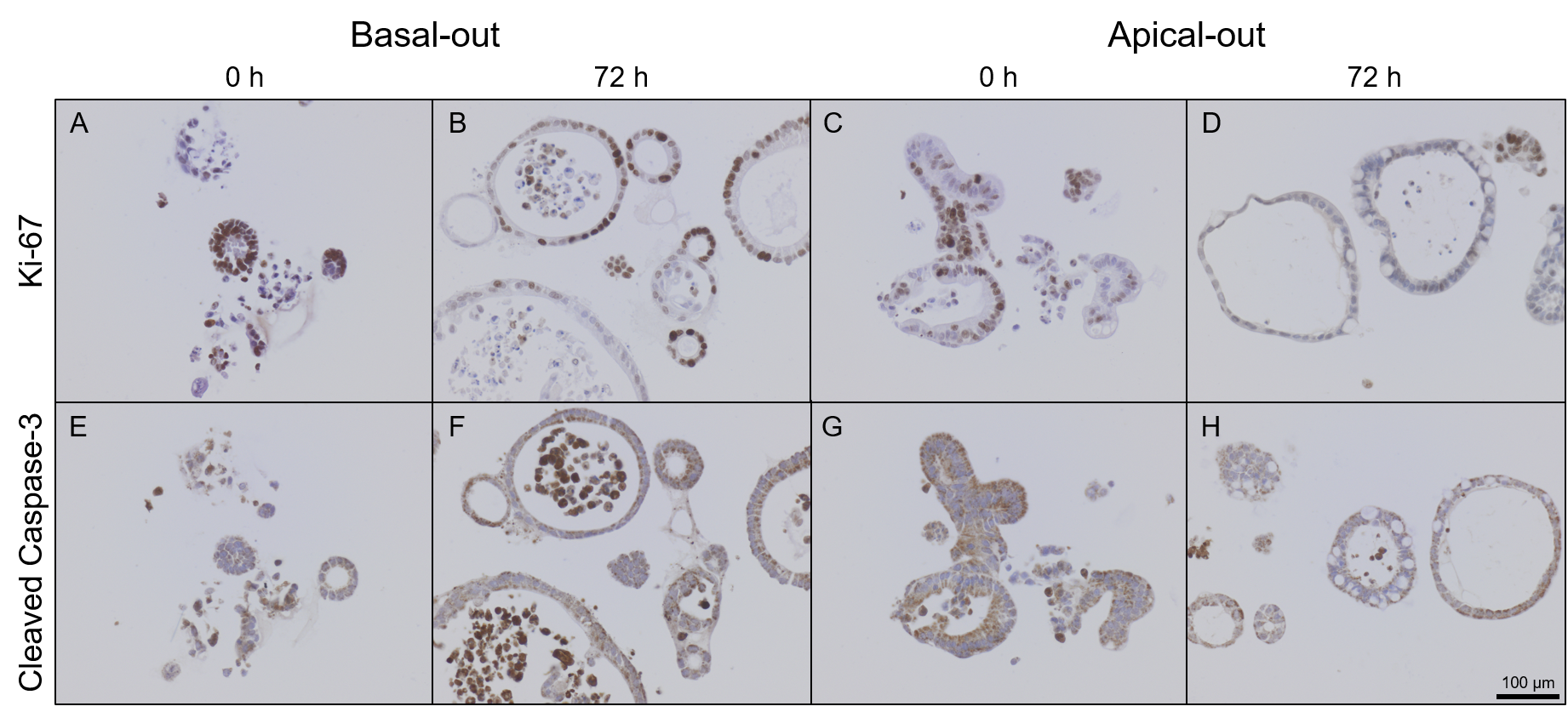


Supplementary figure 3: Immunohistochemical stainings of basal-out and apical-out large intestinal organoids for proliferation marker Ki-67 and apoptosis marker cleaved caspase-3. Scale bar = 100 µm.

Supplementary table 1: Results of statistical analysis of viability, apoptosis and necrosis assays of basal-out vs. apical-out intestinal organoids.

| **Sample** | **Analysis** | **Parameter** | **P value** |
| --- | --- | --- | --- |
| **Small intestine** | Viability | Interaction (Time × Polarity) | <0.001 (****) |
|  |  | Time | <0.001 (****) |
|  |  | Polarity | <0.001 (****) |
|  | Apoptosis | Interaction (Time × Polarity) | <0.001 (****) |
|  |  | Time | > 0.1 (not significant) |
|  |  | Polarity | <0.001 (****) |
|  | Necrosis | Interaction (Time × Polarity) | <0.001 (****) |
|  |  | Time | <0.001 (****) |
|  |  | Polarity | <0.001 (****) |
| **Large intestine** | Viability | Interaction (Time × Polarity) | <0.001 (****) |
|  |  | Time | <0.001 (****) |
|  |  | Polarity | 0.0002 (***) |
|  | Apoptosis | Interaction (Time × Polarity) | 0.0017 (**) |
|  |  | Time | <0.001 (****) |
|  |  | Polarity | > 0.1 (not significant) |
|  | Necrosis | Interaction (Time × Polarity) | <0.001 (****) |
|  |  | Time | <0.001 (****) |
|  |  | Polarity | <0.001 (****) |

Statistical test: two-way ANOVA

Supplementary table 2: Target genes and primer sequences of qPCR assays.

| **Gene** | **Use** | **Forward primer**  **Reverse primer** | **Amplicon length (bp)** |
| --- | --- | --- | --- |
| *CHGA* | Gene of interest | AACCCCACGGAGGCACTTA  GCCAACTCTCACCCCTGTG | 70 |
| *DAP3* | Reference gene | CAGTGGTTGAACAGGGCCTG  AGCGAGGTCCTTCCCCAGA | 151 |
| *ESD* | Reference gene | CCTTGTCGTCATTGCTCCG  CAGTGGCATCCACATAAAATCC | 107 |
| *LGR5* | Gene of interest | CCATCTCTAAGAAAGCTGGACC  CAAGCTCTGTAAGGCATGATTTC | 116 |
| *MUC2* | Gene of interest | TCCTCTACCCTCGTCTACTGC  GACGGGCATGACCAGTTGAA | 149 |
| *VIL1* | Gene of interest | TGCCAACACCAAGAGATTGC  GCAGGAACACATCGTCCTCT | 143 |
